# *QuickStats*: Emergency Department Visit Rates* for Motor Vehicle Crashes,^†^ by Age Group — United States, 2018^§^

**DOI:** 10.15585/mmwr.mm7003a4

**Published:** 2021-01-22

**Figure:**
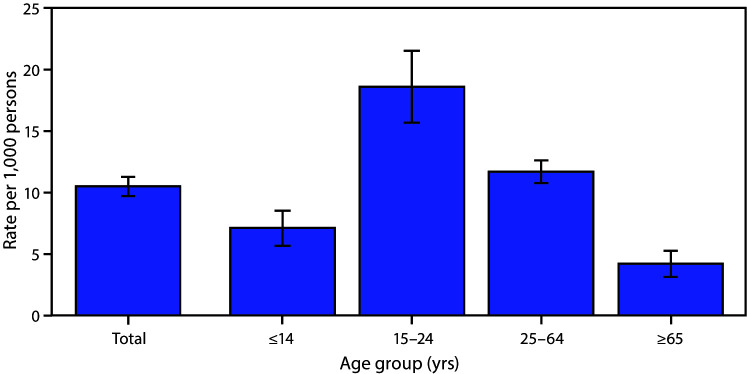
In 2018, the U.S. emergency department (ED) visit rate for motor vehicle crashes was 10.5 visits per 1,000 persons. The ED visit rate for motor vehicle crashes among persons aged 0–14 years was 7.1 ED visits per 1,000 persons. The visit rate for motor vehicle crashes was highest for persons aged 15–24 years (18.6) and declined with age to 11.7 for those aged 25–64 years and to 4.2 for those aged ≥65 years.

**Source:** National Center for Health Statistics. National Hospital Ambulatory Medical Care Survey, 2018. https://www.cdc.gov/nchs/ahcd/ahcd_questionnaires.htm.

For more information on this topic, CDC recommends the following link: https://www.cdc.gov/transportationsafety.

